# Pretreatment of Lignocellulosic Biomass with Ionic Liquids and Ionic Liquid-Based Solvent Systems

**DOI:** 10.3390/molecules22030490

**Published:** 2017-03-20

**Authors:** Qidong Hou, Meiting Ju, Weizun Li, Le Liu, Yu Chen, Qian Yang

**Affiliations:** College of Environmental Science & Engineering, Nankai University, Tianjin 300071, China; hqcd1991@163.com (Q.H.); jumeit@nankai.edu.cn (M.J.); tjliule@126.com (L.L.); chenyu@mail.nankai.edu.cn (Y.C.); 18355302825@163.com (Q.Y.)

**Keywords:** ionic liquids, biomass, dissolution, pretreatment, fractionation, enzymatic saccharification

## Abstract

Pretreatment is very important for the efficient production of value-added products from lignocellulosic biomass. However, traditional pretreatment methods have several disadvantages, including low efficiency and high pollution. This article gives an overview on the applications of ionic liquids (ILs) and IL-based solvent systems in the pretreatment of lignocellulosic biomass. It is divided into three parts: the first deals with the dissolution of biomass in ILs and IL-based solvent systems; the second focuses on the fractionation of biomass using ILs and IL-based solvent systems as solvents; the third emphasizes the enzymatic saccharification of biomass after pretreatment with ILs and IL-based solvent systems.

## 1. Introduction

The search for renewable feedstocks to produce fine chemicals, materials and fuels has become an important goal, with the ever-growing energy demands and environmental concerns, together with the diminishing fossil fuel reserves [1,2]. Lignocellulosic biomass is a promising alternative to fossil resources because of its abundance, renewability and versatility [3]. Lignocellulosic biomass is one of the most abundant renewable resources on the earth and its utilization will not increase net carbon emissions [4,5]. It is anticipated that a sustainable and environmentally friendly organic industry using biomass as a major feedstock will displace the current organic industry which causes serious environmental issues [5]. In recent years, extensive research has established the concept and basic framework of biorefinery, through which biomass can be converted into a series of fuels and process chemicals [1].

Based on the recently developed technologies, biorefinery processes can be categorized into two strategies [6]. One strategy is direct gasification and liquefaction of lignocellulose by pyrolysis. After gasification or liquefaction, the formed products, such as syngas, heat, power, bio-fuels, fertilizer and biochar, can be integrated into the corresponding industrial processes, respectively [6,7,8]. Another strategy is firstly fractionating lignocellulosic biomass into cellulose, hemicelluloses and lignin, and then converting the three biopolymers into some important intermediates and/or final products [9,10]. The latter strategy is well-designed according to the composition and structure of biomass, as it may achieve more complete utilization of biomass and provide more abundant products than the former [10]. Conventionally, cellulose has already been widely used for the production of paper and textiles [9]. From the view of biorefinery, various value-added products can be obtained from cellulose [1]. For instance, bioethanol can be produced via fermentation of glucose, the building unit of cellulose [1,11]. Hemicelluloses are composed of several hexose and pentose sugars, including mannose, galactose, xylose (predominantly) and arabinose. The dehydration product of xylose, furfural, has already been commercialized with an annual production close to 300 kilotons [4]. Lignin is composed of three monomers: coniferyl, sinapyl and pcoumaryl alcohols [12]. Lignin is a promising starting material for the production of aromatic compounds and fuels [13,14,15]. Although many biomass conversion technologies with high potential were developed in the last few decades, few processes are currently in operation owing to many economic and technical barriers [12,16]. The direct conversion of lignocellulose and cellulose usually leads to a relatively low yield and selectivity of products, and the separation and purification of products is also very energy-extensive [6]. The rational design of reaction systems aimed at certain components could obtain more value-added products [17]. Pretreatment to overcome the recalcitrance of lignocellulosic biomass is very important for the efficient and economically viable utilization of lignocellulosic biomass [18,19,20]. However, traditional pretreatment methods have several disadvantages, including low efficiency and high pollution, seriously hindering their large-scale application [10].

Recently, ionic liquids (ILs) and IL-based solvent systems have emerged as promising solvents for lignocellulose, as they provide an effective tool for the pretreatment of biomass [21,22,23,24]. This article gives an overview on the applications of ionic liquids (ILs) and IL-based solvent systems in the pretreatment of lignocellulosic biomass, including the dissolution of biomass, the fractionation of biomass and the enzymatic saccharification of pretreated biomass.

## 2. Dissolution of Lignocellulosic Biomass in ILs and IL-Based Solvent Systems

### 2.1. Dissolution of Cellulose

In 2002, Swatloski et al. reported that IL 1-*n*-butyl-3-methylimidazolium chloride ([Bmim]Cl) can dissolve cellulose with a solubility up to 15% [22]. Since then, numerous studies on the processing of cellulose with ILs have emerged [25]. Up to now, a range of ILs, typically consisting of 1,3-dialkylimidazolium cations and anions with high hydrogen-bond basicity, have been demonstrated as effective cellulose solvents. Readers interested in this respect are referred to several reviews [26,27,28]. As shown in Table 1, the dissolution of cellulose with ILs is influenced by many factors, including the composition and structure of ILs, the nature of cellulose and the dissolution conditions [27].

Many experiments have indicated that the anions of ILs play an important role in determining an IL’s ability to dissolve cellulose [9,29,30]. Up to now, these ILs which are identified as being capable of dissolving cellulose generally consist of imidazolium^+^, pyridinium^+^, ammonium^+^, phosphonium^+^, or morpholinium^+^ based cations, and anions that can form strong hydrogen bonds with hydroxyl groups, e.g., chlorides, carboxylates (acetate, formate, propionate, lactate), dialkyl phosphates, dialkyl and trialkylphosphonates and amino acid anions [26,27]. It is known that the solubility of cellulose in IL corresponds well with the hydrogen-bond basicity of its anion [10,31]. At the same time, the cations can also indirectly influence the dissolving ability of ILs through impacting their physical properties, such as the melting point, density and viscosity [32,33]. It should be noted that the ILs containing anions that can form strong hydrogen bonds are not always capable of dissolving cellulose. For example, the solubility of cellulose in ionic liquid 1-ethyl-3-methylimidazolium acetate ([Emim]Ac) is 8%, while ionic liquid 1-methylimidazolium acetate ([Mim]Ac) is almost unable to dissolve cellulose [9,34]. Therefore, more work is needed to understand the effect of cations on the solubility of cellulose and to reveal the interplay between cations and anions.

Selecting suitable combinations of anions and cations is a promising way to give more efficient and low-cost ILs for (lingo)cellulose dissolution [33,35]. Recently, Mai et al. developed a quantitative structure-activity relationship (QSAR) model to predict cellulose solubility in ILs using the group contribution (GC) and artificial neural network (ANN) methods [33]. Through computer-aided ionic liquids design (CAILD), they screened an optimal IL with a cellulose solubility at least 1.2 times higher than that of [DEME][MEPA], one of the best ILs reported for cellulose dissolution. Liu et al. evaluated the cellulose-dissolving ability of 357 ILs formed from 17 cations and 21 anions by COSMO-RS (conductor-like screening model for real solvents) [26]. These results suggested that the suitable ILs for cellulose dissolution consist of cations including methylimidazolium^+^, pyridinium^+^, ethylmorpholinium^+^ and methylpyrrolidinium^+^ which can be functioned by ethyl, allyl, 2-hydroxylethyl, 2-methoxyethyl and acryloyloxypropyl, and anions including Ac^−^, Dec^−^ (decanoate), HCOO^−^, Cl^−^, BEN^−^ (Benzoate), DMPO_4_^−^ (dimethylphosphate), DEP^−^ (diethylphosphate), DBP^−^ (dibutylphosphate) and Br^−^. Abe et al. systematically investigated the influences of the polarity, hydrophobicity, and density of ILs on cellulose solubility and they found that the elongation of the alkyl groups on the phosphonium cations considerably impacts both the hydrophobicity and cellulose-dissolving ability of the ILs [36]. It was also observed that the density of ILs significantly impacts their cellulose-dissolving ability. Based on this, they succeeded in synthesizing a series of ILs which are not only capable of dissolving cellulose, but also separable with water at moderate temperatures.

Solvent systems consisting of ionic liquids and co-solvents are being recognized as excellent alternative solvents for the dissolution and processing of cellulose. Compared with the parent ILs, some solvent systems showed obvious advantages, including a higher dissolution rate, raised thermal stability, and reduced viscosity [37]. Rinaldi et al. found that the solvent systems consisting of aprotic polar solvents, such as *N*,*N*-dimethylformamide (DMF), dimethylsulphoxide (DMSO), *N*,*N*-dimethylacetamide (DMA) and 1,3-dimethyl-2-imidazolidinone (DMI), and a small molar fraction of ionic liquid could dissolve large amounts of cellulose instantaneously at relative low temperatures (50 °C), while the dissolution of cellulose in pure ionic liquid requires relatively high temperatures and a long time [38]. In addition, Xu et al. designed highly effective cellulose solvent systems by adding DMSO to 1-butyl-3-methylimidazolium acetate ([Bmim]Ac) [39]. Both microcrystalline and cotton with a high degree of polymerization can be directly dissolved in these solvent systems at ambient temperature (25 °C) without heating. They attributed the improved dissolving ability to the preferential solvation of the cations of the IL by the aprotic polar solvent, as is supported by the conductivity measurements. Tao et al. found that DMSO could not only promote the dissolution of cellulose in ILs, but could also show a superior cellulose-protective effect [40]. The degree of polymerization (DP) of cellulose was unchanged after dissolution and regeneration from the solvent system [Emim]Ac/DMSO, while the obvious reduction of the DP of cellulose was observed in pure IL. These IL-based solvent systems have the potential to maintain (or even to improve) moderate-to-high cellulose solubility, while simultaneously minimizing problems associated with handling highly viscous solutions of cellulose in pure ILs. The influence mechanism of DMSO on the cellulose-solvating ability of 1-allyl-3-methylimidazolium chloride ([Amim]Cl) was investigated through excess infrared spectroscopy, nuclear magnetic resonance (NMR) T_2_ relaxometry, ^1^H-NMR, ^35^Cl-NMR and dynamic light scattering [41]. The results showed that when the DMSO concentration is low, the tight association between the cation and anion in the [Amim][Cl] network is disrupted and the mass transport is accelerated, thus enhancing the dynamics of [Amim][Cl]. As the molar fraction of DMSO further increased to a certain extent, ion clusters started to form, leading to the decreased ability of [Amim][Cl] to form hydrogen bonds with cellulose. Therefore, the cellulose solubility in the [Amim][Cl]/DMSO mixture decreased when the molar fraction of DMSO exceeded 0.5. Besides, Xu et al. found that adding 1.0 wt. % of lithium salt (LiCl, LiBr, LiAc, LiNO_3_, or LiClO_4_) into [Bmim]Ac could also significantly increase the solubility of the cellulose [42]. The improved solubility of cellulose was due to the disruption of the intermolecular hydrogen bond through the interaction of Li^+^ with the hydroxyl oxygen O(3) of cellulose, as was suggested by the ^13^C-NMR spectra. The use of a dispersing agent, including acetone, acetonitrile, DBN or DMSO, could not only promote the dissolution of cellulose in 1,5-diazabicyclo(4.3.0)non-5-enium acetate ([HDBN][OAc]^−^)–based solvent systems, but also could improve the acetylation process significantly [43]. Besides, γ-valerolactone (GVL), a renewable solvent derived from biomass, could also serve as a co-solvent for the dissolution of cellulose in ILs [44]. Although GVL was marginally less efficient than DMSO, GVL was significantly safer than DMSO.

Adding anti-solvents, such as water or ethanol, to the solution can precipitate cellulose from ILs. The regenerated cellulose can be obtained via filtration or centrifugation [27]. To recover IL, the removal of the anti-solvent from the IL by distilling is generally needed. This is an energy-intensive process which may bring a series of economic and engineering challenges in practice. Barber et al. attempted to use supercritical carbon dioxide (scCO_2_) to displace traditional anti-solvents [45]. They found that the amount of the IL needed to be recycled through distilling was reduced by 66% after precipitating biomass with scCO_2_. Although completely avoiding the use of water seems impossible, the partial displacement of water with CO_2_ could considerably improve the economic feasibility of the whole process. It has been reported that cellulose can drive the phase separation of cellulose solutions in the solvent system consisting of [Emim]Ac and 1,3-dimethyl-2-imidazolidinone (DMI) [37]. The temperature of the phase separation can be regulated through adjusting the mole fractions of the IL, co-solvent, and cellulose, as well as providing a new perspective for the design of cellulose regeneration and solvent recycling strategies.

### 2.2. Dissolution of Lignin

The solubility of lignin in ILs is an important parameter for the design of IL-based biorefinery processes [48]. The dissolution of lignin is generally tested using kraft lignin, alkaline or organosolv lignin, which are commercially available lignin products produced in the chemical pulping process, as model lignins [49]. It should be noted that the solubility of these lignin models cannot precisely reflect their exact dissolving ability to become lignin from biomass since both the composition and structure of lignin varies in different biomasses [10]. The solubilities of lignin in some representative ILs and ILs-based solvent systems are shown in Table 2. The ILs with moderate hydrogen-bonding anions seem to be more capable solvents for lignin. Rashid et al. synthesized three protic ionic liquids, including pyridinium formate ([Py][For]), pyridinium acetate ([Py][Ac]) and pyridinium propionate ([Py][Pro]), and investigated their performance for the dissolution of lignin [48]. They found that the three ionic liquids could dissolve a large amount of lignin in a wide range of temperatures (20–140 °C). The solubility of lignin at 75 °C is 70 wt. %, 64 wt. % and 55 wt. % for [Py][For], [Py][Ac] and [Py][Pro], respectively. Achinivu et al. reported that [Py][Ac] could selectively dissolve lignin at 75 °C, while 1-methylimidazolium acetate ([Mim][Ac]) and pyrrolidinium acetate ([Pyrr][Ac]) could dissolve lignin and xylan but could not dissolve cellulose [34]. They also investigated the influence of water on the lignin solubility. Although increasing the water content resulted in the decrease of the lignin solubility, the solubility was still more than 20 wt. % when the water content did not exceed 20%. Under the same conditions, the solubility of cellulose in [Py][For] was less than 1 wt. %, suggesting [Py][For] is a selective solvent for lignin. It was observed that [Mmim][MeSO_4_] and [Bmim][CF_3_SO_3_] can dissolve more than 50 wt. % lignin at relative low temperatures, but they are not capable of dissolving cellulose even at elevated temperatures [49,50]. Yan et al. found a series of amine-sulfonate functionalized ionic liquids (ASF-ILs) can dissolve kraft lignin and lignosulfonate efficiently at 373 K, but cannot dissolve xylan and cellulose at the same conditions (solubility < 0.5%) [51]. Mu et al. reported that a series of solvents consisting of *N*-methyl-2-pyrrolidone (NMP) and mono/di-carboxylic acids are capable of dissolving lignin with a lignin solubility as high as 60 wt. % [52].

Xue et al. reported that the solvent systems GVL/[Bmim]Ac and GVL/[Amim]Cl are more capable of dissolving lignin compared with the corresponding parent IL [53]. Moreover, the dissolution of lignin is more tolerant to water than the dissolution of cellulose. The high solubility of lignin in the mixture of ILs and water can be achieved, while the presence of water has a remarkably negative impact on the dissolution of cellulose in ILs [54]. Wang et al. investigated the influence of water on the dissolution of lignin in the IL-water mixtures at 60 °C using various cations, anions and IL contents. They found that the addition of the appropriate amount of water to [Emim]Ac can significantly increase the lignin solubility. The highest solubility of lignin (45 wt. %) was achieved with an IL content of 70 wt. %. The improved dissolution of lignin was attributed to the increased interaction probability between lignin and “free” ions, whose amount and mobility were remarkably increased after the addition of water.

### 2.3. Dissolution of Lignocellulose

Based on the capability to dissolve lignin, cellulose and hemicelluloses, ILs can be grouped into five types: (1) unselective ILs that can dissolve lignin, cellulose and hemicelluloses simultaneously; (2) ILs that can selectively dissolve lignin and hemicelluloses but cannot dissolve cellulose; (3) ILs that can selectively dissolve hemicelluloses but cannot dissolve cellulose and lignin; (4) ILs that can selectively dissolve lignin but cannot dissolve cellulose and hemicelluloses; and (5) ILs that cannot dissolve cellulose, lignin or hemicelluloses. The first, second, fourth and fifth types of ILs have already been reported previously, but the third type of IL has not been proved in practice, to the best of our knowledge.

Casas et al. investigated the correlation between the differential solubility of cellulose and lignin in ionic liquids and activity coefficients [50]. Based on the solubility of cellulose and lignin in a representative sample of 12 ILs, a reliable COSMO-RS computational approach was developed to predict the ability of an IL to dissolve cellulose and/or lignin. Janesko et al. investigated the interactions of ionic liquid with cellulose and lignin by dispersion-corrected density functional theory (DFT-D), using (1,4)-dimethoxy-β-d-glucopyranose and 1-(4-methoxyphenyl)-2-methoxyethanol as models for cellulose and lignin polyphenols, respectively [55]. They found that the cellulose model predominantly interacts with anions, whereas significant interactions between the lignin model and the imidazolium cations were observed. These results suggested that the different solubility of lignin and cellulose in the same IL is mainly results from the different dissolution process [55,56]. The selective dissolution of xylan, the most predominant type of hemicellulose, was also attempted by Froschauer et al. by modifying the anion of IL 1-butyl-3-methylimidazolium dimethyl phosphate via substituting one oxygen atom with sulfur and selenium [57]. They found that reducing the hydrogen bond basicity of the IL could suppress the dissolution of cellulose, while the dissolution of xylan was almost uninfluenced, as seems to be similar with the dissolution feature of lignin in selective ILs. Unfortunately, the comparison of the dissolution of lignin and xylan cannot be performed due to the lack of data on the dissolution of lignin in this study.

Generally speaking, ionic liquids that can dissolve cellulose are also capable of dissolving lignocellulose [58,59]. Fort et al. investigated the dissolution of wood chips in [Bmim]Cl [21]. The dissolution process of wood in [Bmim]Cl was observed through NMR analysis and they found that the weight ratio of the dissolved cellulosic material to lignin across the whole dissolution profiles was largely constant at 2:1, which is consistent with the original composition of the biomass, demonstrating that the dissolution of cellulose and lignin occurs simultaneously without obvious selectivity. However, complete dissolution was not achieved, even at prolonged times, probably due to the relatively large particle size of the wood chips (~5 mm × 5 mm in size). Subsequently, Kilpel et al. reported that after ball-milling, the complete dissolution of both hardwoods and softwoods can be realized in [Bmim]Cl and [Amim]Cl [60]. After several hours’ treatment, no fibrous material was observed through the hot-stage optical microscopy, demonstrating the complete dissolution of biomass in IL. They found that reducing the particle size of the biomass can significantly accelerate the dissolution, but the presence of water will significantly reduce the solubility of wood.

Sun et al. found that the use of polyoxometalate catalysts, which can selectively cleave lignin-carbohydrate bonds, would enhance the dissolution and decrease the lignin content in the recovered pulp [61]. These results suggested that the dissolution of polysaccharides which are not bonded to lignin or with fewer lignin bonds is easier than the dissolution of the supramolecular network formed by the three biopolymers through complex entanglements and covalent linkages. The structure of switchgrass dissolved in IL [Emim]Ac was investigated by small-angle neutron scattering (SANS) data and the results showed that the entangled cellulose-hemicellulose-lignin network formed in plants cannot be completely disrupted in the dissolution process [62]. Sun et al. observed the changes of the wall morphology and chemical compositions of wood during IL pretreatment at the cell level via hyperspectral confocal fluorescence microscopy and Raman microscopy [63]. They found that the dissolution of lignin by [Emim]Ac occurs rapidly in the secondary cell walls, while the dissolution of cellulose occurs with no preference in different layers or regions of cell walls. It was also observed that the dissolution rate of cellulose is higher than that of lignin despite the crystallinity of cellulose.

The presence of water in IL is usually detrimental to the dissolution of biomass. Polar ionic liquids can readily absorb water from the atmosphere since they are highly hygroscopic. It is very difficult to fully dehydrate IL and biomass in practice. Therefore, developing a solvent system consisting of water and IL, which is capable of dissolving lignocellulose without a drying process, is very attractive. Pang et al. designed a promising solvent system composed of [Bmim]Cl, water, and lithium chloride for the dissolution of lignocellulose (bamboo) [64]. They found that the tolerance of [Bmim]Cl to water was significantly enhanced by the addition of lithium chloride. The dissolution rate of bamboo (5 wt. % loading with respect to the solvent system) in the solvent system consisting of 45 wt. % BmimCl and 55 wt. % LiCl·2H_2_O (25 wt. % overall water content in the solvent system) is approximately 80%. In the dissolution process, lignin and hemicelluloses were selectively dissolved by 96% and 92%, respectively, while the undissolved residue mainly consisted of cellulose (~86%) with a small amount of lignin (<5%). Abe et al. achieved the almost complete dissolution of woody biomass at mild conditions (60 °C, 24 h) using tetra-*n*-butylphosphonium hydroxide ([P_4,4,4,4_]OH) aqueous solution and H_2_O_2_ [65]. In the absence of H_2_O_2_, only partial dissolution of hard woods (oak and eucalyptus) and soft woods (pine, spruce) was achieved in the neat [P_4,4,4,4_]OH aqueous solution. They found that the increase of Klason lignin content in the wood samples results in the decrease of their dissolution degrees in [P_4,4,4,4_]OH) aqueous solution. When a suitable amount of H_2_O_2_ was added, almost complete dissolution of woody biomass could be achieved regardless of the wood species. However, the decrease of the molecular weight of the cellulose was also observed as a consequence of the treatment with [P_4,4,4,4_]OH)/H_2_O_2_ aqueous solution. Boissou et al. reported that the ionic liquids consisting of levulinate anions and short-chain alkyl ammonium cations could tolerate the presence of up to 18 wt. % of water [66]. Moreover, the addition of 20 wt. % of GVL to such ILs could increase the cellulose solubility to 20 wt. % while simultaneously improving the sustainability of these media.

Based on the dissolution ability of lignocellulosic biomass in IL, Sun et al. developed an efficient process to directly produce composite fibers from southern yellow pine and bagasse dissolved in [Emim]Ac through a dry-jet wet spinning method [67]. They found that the dissolution of bagasse using short dissolution times (10–30 min) and temperatures above the glass transition temperature of lignin could give fibers stronger than those obtained using the lower temperature/longer time. Only when pine was dissolved at a high temperature/short time, fibers could be spun from the obtained solutions, with a lower stress value than that obtained from bagasse.

## 3. Fractionation of Lignocellulosic Biomass with ILs and IL-Based Solvent Systems

**Figure 1:**
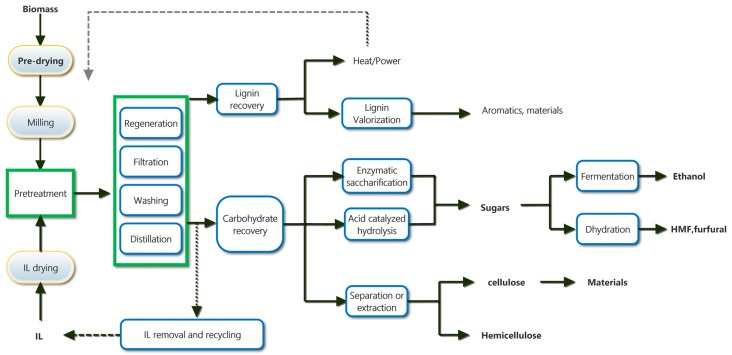
Typical routes for the pretreatment of lignocellulosic biomass with ILs.

Typical routes for the pretreatment of lignocellulosic biomass with ILs are summarized in Figure 1. Owing to the different dissolution characteristics of cellulose, hemicelluloses and lignin in ionic liquids, both the composition and structure of biomass will change when the biomass is treated with ionic liquids. These changes could promote the fractionation of lignocellulosic biomass into cellulose, hemicelluloses and lignin, and then enhance the subsequent upgradation of these fractions, especially the enzymatic saccharification of treated biomass [68]. In this section, we discuss the fractionation of lignocellulosic biomass with ILs and IL-based solvent systems.

### 3.1. Separation and Recovery of Lignin from Biomass

The lignin separated from lignocellulosic biomass is not only a desirable renewable feedstock for the production of fuels and fine chemicals, especially aromatic compounds, but also a promising starting material for the production of novel materials [69]. For example, Qian et al. reported that lignins are high-performance broad-spectrum sunscreens, providing a promising alternative to the currently used synthetic sunscreen actives [70]. Therefore, the extraction of lignin from biomass could greatly improve biorefinery productivity and profitability [68].

**Figure 2:**
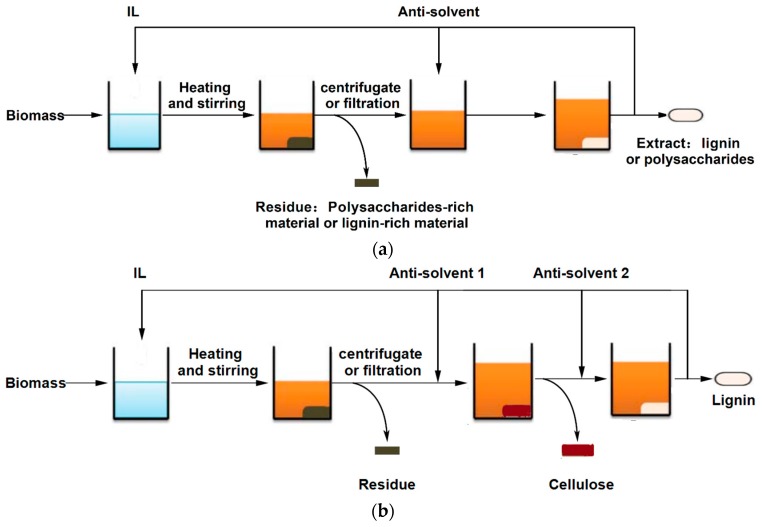
Fractionation procedure of lignocellulose with ILs: (**a**) one-step extraction of lignin or polysaccharide using selective ILs and (**b**) two-step separation of cellulose and lignin with non-selective ILs. Adapted from Reference [73] with permission from The Royal Society of Chemistry.

As mentioned above, some ILs can selectively dissolve lignin while cellulose is almost insoluble in these ILs. These ILs may enable the direct extraction of lignin from lignocellulosic biomass (Table 3). Pinkert et al. reported the selective extraction of lignin from wood biomass, including *Pinus radiata* and *Eucalyptus nitens*, using ILs [Bmim]Ace and [Emim]Ace [71]. These two ILs were synthesized by substituting the anions of [Bmim]Cl and [Emim]Cl with acesulfamate ions (Ace^−^). The large and bulky anions with a delocalized charge enable the two ILs that are unable to dissolve cellulose. The procedure of extracting lignin from biomass using these two ILs is shown in Figure 2a. The mixture of wood flour and IL was heated and stirred at the desired conditions to selectively dissolve the lignin and then the insoluble residues were separated from the solution by filtration. The dissolved lignin was precipitated from the solution upon the addition of acetone and then separated by filtration. After the removal of acetone by distilling, the IL was recycled and reused for lignin extraction. Up to 43% and 60% of wood lignin can be extracted using pure IL and a solvent system consisting of IL and DMSO, respectively. The characterizations indicated that the extracted lignin had a larger molar mass and a more uniform molar mass distribution than kraft lignin. Moreover, the crystallinity of cellulose in the cellulosic-rich residues was preserved due to the insolubility of cellulose in these solvents. The obtained lignin and cellulosic-rich wood residue seems to be a promising feedstock for the manufacturing of novel composite materials. However, Abushammala et al. reported that when spruce wood is treated with [Bmim]Ace, side reactions, especially the degradation of the acesulfamate anions into sulfate, dominate the delignification process, and may limit the practical application of these ILs [72].

Achinivu et al. developed a highly effective method to extract lignin from lignocellulosic biomass using protic ionic liquids, including [Py][Ac], [Mim][Ac] and [Pyrr][Ac] [34]. As described above, the solubility of lignin in the three ILs is high, while cellulose is almost insoluble in them. This property enables the selective dissolution of lignin from lignocellulosic biomass. When corn stock was treated at 90 °C for 24 h, more than 70% of the lignin was extracted and a polysaccharide-rich stream was left after the extraction step. The lignin extraction efficiency increased in the order [Py][Ac] < [Mim][Ac] < [Pyrr][Ac], as is directly proportional to solubility of xylan in the PILs. Therefore, they postulated that the improved dissolution of xylan in the PIL promotes the disruption/penetration of fiber in the biomass and then significantly enhances the dissolution and extraction of lignin. Although a few sugars were extracted simultaneously with the lignin during the dissolution process, they can be readily separated from the lignin by washing with water.

Yan et al. demonstrated that the amine-sulfonate functionalized (ASF) ILs, which are capable of dissolving lignin but do not dissolve xylan and cellulose, are efficient for the fractionation of eucalyptus bark [51]. More than 40% of the lignin was selectively extracted by the ASF-ILs at 393 K for 10 h and simultaneously the enzymatic saccharification of the residue to sugars was also considerably improved. Muhammad et al. investigated the extraction of lignin from bamboo biomass using nitrile-based ILs, including 1-propyronitrile-3-butylimidazolium chloride ([C_2_CNBim]Cl), 1-propyronitrile-3-allylimidazolium chloride ([C_2_CNAim]Cl), 1-propyronitrile-3-(2-hydroxyethyl)imidazolium chloride ([C_2_CNHEim]Cl) and 1-propyronitrile-3-benzyllimidazolium chloride ([C_2_CNBzim]Cl) [77]. Among the tested ILs, [C_2_CNBzim]Cl was found to be the most efficient, successfully extracting 53% of the lignin from the bamboo.

Anugwom et al. developed an efficient process to remove lignin from Nordic woody biomass using a highly diluted, aqueous ‘SO_2_-switched’ switchable ionic liquid (SIL) based on an alkanol amine (monoethanol amine, MEA) and an organic superbase (1,8-diazabicyclo-[5.4.0]-undec-7-ene, DBU) [78,79,80]. They found the solvent system is selective towards lignin dissolution and substantial removal of lignin could be achieved at relatively low amount of SIL. Meanwhile, cellulose-rich pulp with a low lignin content, complemented by hemicelluloses, could be obtained [81]. The SIL was also capable of separating carbohydrate-free lignin from technical lignin, such as sodium lignosulfonate [82]. It was reported that loop reactor systems could improve the dissolution of hemicelluloses and lignin by facilitating the heat and mass transfer, compared with the traditionally used batch reactor [83]. Besides, it was also found that stirring could decrease the condensation of lignin, increase the dissolution efficacy and then enhance the wood fractionation [84].

Hiltunen et al. demonstrated that a solvent system consisting of boric acid and choline chloride could be used as an efficient solvent for biomass fractionation [85]. After filtrating the formed solution of biomass treated by this solvent system, lignin-rich material was obtained from the smallest fractions. This result suggested that this solvent dissolved lignin more readily than other biopolymers. The characterization indicated this lignin is non-condensed lignin which may be suitable for special uses. An et al. pretreated rice straw with cholinium ionic liquids that are wholly composed of biomaterials [86]. The biomass was fractionated into carbohydrate-rich materials (CRMs) and lignin-rich materials (LRMs) in the selective precipitating process through adjusting the pH. Approximately 46% of the lignin in the native rice straw was fractionated as LRM after being treated by cholinium argininate ([Ch][Arg]). Cheng et al. found that [Cho][Ac] could selectively dissolve hemicellulose and lignin from ground bagasse or southern yellow pine [87]. After the dissolution, the undissolved cellulose pulp with reduced lignin content was obtained. The pulp was further treated with [Emim]Ac and then regenerated as cellulose-rich material (CRM) with a lignin content (10.6%) significantly lower than that of the original bagasse (23.6%). Eta et al. reported that CO_2_- and CS_2_-based switchable IL could remove 64 wt. % of hemicelluloses and 70 wt. % of lignin from Nordic hardwood (Betula pendula), leaving a cellulose-rich fraction [88].

Rodriguez et al. found that gaseous NH_3_ and O_2_ could enhance the removal of lignin from miscanthus dissolved by [Emim]Ac [89]. The materials treated with IL in the presence of NH_3_ and O_2_ had a lignin content lower than 10%, whereas that treated in air or N_2_ had a lignin content of ca. 20%. The cellulose content in the regenerated materials in the former was also higher than the latter. Vo et al. developed a process to separate carbohydrates from poplar wood sawdust using a mixture solvent consisting of IL [Bmim]Ac and 1,3-dimethylimidazolium methyl phosphite ([Dmim][MeO(H)PO_2_]) [90]. The wood was fractionated into three types of products: the water-soluble fraction, the water-insoluble fraction and the IL-dissolved fraction. The water-soluble fraction was found to consist predominantly of carbohydrate (81%) with a low lignin content (3.5%), while the 55% of the native lignin of the original biomass remained in the water-insoluble fraction. It was also observed that the phosphorylation of both carbohydrates and lignin increases along with the increase of the dissolution temperature and the time.

The solvent systems consisting of IL and other solvents can be also used for the extraction of lignin from biomass. Liang et al. treated bamboo powder with an optimized ionic liquid (BmimBr)–ethanol–water mixture (38:38:24 in volume ratio) with the liquid/solid ratio of 10 mL/g at 170 °C for 4 h [91]. The resulting mixture was washed with ethanol and filtrated and then the cellulose fraction with a purity of 95.2%–96.1% was obtained as residue (45.7 wt. %–46.2 wt. % of the original biomass), whereas most of the lignin (95.7 wt. %–96.9 wt. % of original lignin) and hemicellulose (97.0 wt. %–97.8 wt. % of original hemicellulose) fraction was dissolved in the filtrate. The residual lignin in IL solutions was removed by ultrafiltration and then the IL was recovered by electrodialysis.

The structural features of lignin macromolecules (IL-L) extracted from poplar wood (*Populus albaglandulosa*) with ionic liquid were compared with the corresponding milled wood lignin (MWL) by Kim et al. [92]. They found that the content of the functional groups (OMe and phenolic OH) were similar for the two lignin samples, although the yield of the former was higher than the latter. In addition, both the average molecular weight (6347 Da vs. 10,002 Da) and the polydispersity index (1.62 vs. 2.64) of IL-L were lower than of that of MWL, suggesting that the lignin was disrupted into more uniform fragments during the IL dissolution process.

Except for the direct extraction of lignin from biomass, the IL-based method can be also used for the separation of cellulose and lignin to obtain high-purity cellulose and lignin. Pang et al. developed a green bleaching method through extracting residual lignin from kraft pulp using aqueous ILs under benign conditions [93]. Although the extraction rate of lignin is less than 30%, this process can significantly improve the bleaching properties of pulp without degrading cellulose. As an alternative to the traditional bleaching process, this method can reduce the usage of bleaching chemicals and the toxicity of the bleaching effluent, and then avoid the concomitant environmental issues.

### 3.2. Separation and Recovery of Cellulose-Rich Materials from Biomass

Abe et al. first reported the direct extraction of polysaccharides from bran using a series of 1-alkyl-3-methylimidazolium phosphonate and phosphinate-type ionic liquids [94]. They found that the IL 1-ethyl-3-methylimidazolium phosphinate could selectively dissolve the polysaccharides in bran at mild temperatures (25–50 °C). Subsequently, Abe et al. reported that the solvent system consisting of tetra-n-butylphosphonium hydroxide and water is also capable of extracting polysaccharides from wood [73]. The extracted material consisted mainly of cellulose (glucan) and xylan, while the lignin was mostly left in the undissolved residue rather than in the extract [73]. The solvent system containing 40 wt. % water was employed because this solvent system could dissolve pure cellulose without heating. After gentle stirring at room temperature for only 1 h, 37% of the polysaccharides from woody biomass were extracted. When the treatment was prolonged to 24 h, the extent of the polysaccharide extraction reached up to 62%. Since the dissolution ability of the solvent system can be maintained in a wide range of water contents, the extracting performance is unaffected by the small amount of water that exists in the biomass after air-drying. Moreover, the solvent system can be concentrated in the open air and then automatically recover the dissolution ability, as this will significantly reduce the energy consumption. Pezoa-Conte et al. reported that the distillable ionic liquid [TMGH^+^][EtCO_2_^−^] (1,1,3,3-tetramethylguanidine propionate), which is synthesized by a simple acid-base neutralization reaction, could extract up to 67 wt. % of carbohydrates from the green alga *Ulva rigida* [95].

Non-selective ILs could also be used to obtain cellulose-rich material and lignin through a two-step separation process, as shown in Figure 2b. The woods, including pine, poplar, eucalyptus, and oak, were treated with the solvent system consisting of IL [Bmim]Cl and DMSO-*d*_6_ (15 wt. %) [21]. The ^13^C-NMR spectrum showed 44 wt. % of the cellulosic materials present in pine wood could be extracted by this solvent system in 12 h. It was also found that the ratio of dissolved cellulosic material to lignin was largely constant and correlated well with the original chemical composition of the wood, suggesting that the solvent system dissolves the two polymers from the wood fibers simultaneously without obvious selectivity. However, when some precipitating solvents, including the 1:1 acetone–water mixture, dichloromethane or acetonitrile, were added to the resulting liquors, celluloses which are virtually free of lignin and hemicellulose were obtained. Since both lignin and hemicelluloses were present in the IL-based wood liquors before the cellulose precipitation step, they postulated that the separation of cellulose with other biopolymers is achieved through the selective precipitating process. After the cellulose precipitation step, the lignin and hemicelluloses were still dissolved in the solution, as was confirmed by the ^13^C-NMR spectra. The particle size of the biomass also has a significant impact on the fractionation performance. Leskinen et al. reported that a cellulose-rich material with a relatively low (6.2%) lignin content can be obtained from Norway spruce wood with approciate particle size tuned by preparatory milling [96,97].

Cellulose obtained from different approaches may be suitable for different applications. For instance, narrowly distributed cellulose with a high molecular weight is very attractive for material applications and derivatizations, especially if the native cellulose I crystal structure is preserved, while low-molar-mass celluloses and cello-oligosaccharides (COS) also have several potential applications, such as prebiotics in food products [98]. Shen et al. prepared physical and/or covalently linked (chemical) hydrogels from cellulose extracted with ionic liquid from wood biomass, respectively, and compared them with the hydrogels prepared from commercially available microcrystalline cellulose [99]. They found that the IL-extracted cellulose pulp could form both stable physical and chemical hydrogels by initial dissolution of the biopolymers in NaOH/urea aqueous systems using freeze/thaw cycles, followed by thermal treatment and supercritical CO_2_ drying, whereas biopolymers of a lower apparent molecular weight, such as microcrystalline cellulose, cannot form a hydrogel without a covalent cross-linker. Moreover, hydrogels prepared from the IL-extracted cellulose showed unique properties which were different from those made from the commercially available cellulose.

### 3.3. Separation of Cellulose and Hemicelluloses

Ionic liquids can be also used to separate cellulose and hemicelluloses. High-purity cellulose, such as dissolving pulp, is useful for many applications, including the production of cellulosic fibers and films. Paper-grade pulp could serve as a cheap feedstock for the production of high-purity cellulose. Since paper-grade pulp is rich in hemicellulose, a refining process to separate cellulose and hemicelluloses is necessary to give high-purity cellulose. Froschauer et al. developed an environmentally benign fractionation method (IONCELL-P method) using a solvent system consisting of a co-solvent (water, ethanol, or acetone) and [Emim]Ac to extract hemicelluloses from paper-grade pulp [100]. This ratio of IL and co-solvent was optimized according to the Kamlet-Taft parameters. After stirring at 60 °C for 3 h, the paper-grade kraft pulp was fractionated into a separated cellulose and a regenerated hemicellulose fraction. The obtained cellulose and hemicelluloses exhibited high purity. Besides, little cellulose and hemicellulose was lost in the fractionating process. This process provides an environmentally friendly and economically efficient method for the production of high-purity dissolving pulp. Stepan et al. investigated the IONCELL-P fractionation process using cotton linters as a model cellulose substrate [98]. They found that the cotton linters after ozone treatment can be fractionated into pure cellulose and hemicellulose fractions almost quantitatively using the solvent system consisting of [Emim]Ac and water.

### 3.4. Extraction of Other Components from Biomass

Except for the fractionation of lignocellulose, ILs are also promising for the extraction of other components from biomass, especially those containing specific high-value components [101]. For instance, microalgae have attracted much attention not only as one of the most promising sources of sustainable biomass, but also as a renewable feedstock for the production of biodiesel due to the high content of lipids [102]. However, the collection and desiccation of microalgae are energy-intensive and consequently costly, as this will be one of the largest challenges for the utilization of microalgae. Compared with traditional organic solvents–based lipid extraction methods which require the complete drying of microalgae, functionalized ionic liquids may allow the direct extraction of lipids from wet biomass. Pan et al. reported that IL 1-butyl-3-methylimidazolium hydrogen sulfate ([Bmim][HSO_4_]) could extract lipids without drying from three algal species, including *Chlorella sorokiniana*, *Nannochloropsis salina* and *Galdieria sulphuraria* [103]. They also found that microwave irradiation and mineral acids could greatly improve the extraction rate when compared with the conventional solvent extraction method. Young et al. investigated the extraction of bio-oils from *Chlorella* microalgae using solvent systems consisting of 1-ethyl-3-methyl imidazolium methyl sulfate ([Emim][HSO_4_]) and several organic solvents, including methanol, dimethyl sulfoxide, acetic acid, methanol, acetone, and chloroform [104]. It was observed that methanol could give a lipid yield approaching the realistic lipid content in biomass, while acetone and chloroform give higher yields of products but with lower purity. Chen et al. developed an ionic liquid-assisted subcritical water method for the extraction of lipids from wet microalgae *Scenedesmus* sp. [105]. They found that the optimal lipid yield was obtained at 110 °C with a 1% concentration of the ionic liquid [HNEt_3_][HSO_4_]. Under these conditions, the lipid yield (35.67%) was comparable to that of the Bligh and Dyer method (35.32%), with a triacylglycerol content (73.63%) significantly higher than that of the traditional method (60.44%). Moreover, the algae cell residues assembled into microspheres during the extraction process, readily allowing their separation from the solvent. It should be noted that more work is needed to recover the downstream fractions, such as carbohydrates and carotenoids, to achieve the ful utilization of algal biomass.

### 3.5. Stability and Reuse of Ionic Liquid

The recovery and reuse of ILs is very important for the practical employment of IL-based fractionation technology. However, the instability of ILs and the possible contamination resulting from the reactions between ILs and substrates are adverse for their reuse. For instance, Clough et al. investigated the stability of carbohydrates dissolved in ILs at temperatures (100–120 °C) which are comparable to those of industrial cellulose reprocessing protocols [106]. They found that carbohydrates dissolved in [Emim]Ac, one of the most widely used ILs for processing biomass, and underwent a series of decomposition reactions, yielding a terminal adduct species, 1-ethyl-2-(hydroxymethyl)-3-methylimidazolium acetate. The formation of adducts was also observed when other carboxylate ILs were used. In contrast, they found that [Bmim]Cl did not react with carbohydrates. Clough et al. found adding small molar quantities of glycerol to carboxylate ILs could significantly suppress the formation of adduct accumulation and enable their long-term thermal stability and recyclability [107].

The recovery of common ILs is generally done through distilling anti-solvents from ILs under vacuum. However, this process is usually energy-extensive, thus greatly limiting their practical application. An et al. reported the total recovery rate of renewable cholinium ILs was 75% after treating rice straw for eight cycles, suggesting almost 25% of the IL was lost [86]. Since the protic ionic liquids (PILs) synthesized from the acetic acid and base through proton exchange can decompose into the corresponding acid and base at high temperatures, the three PILs can be recovered by decomposition and resynthesis. Almost full recovery of PILs was achieved with this process, suggesting the thermal degradation of PILs into amide by-products did not occur in these conditions [34]. Liang et al. developed a hybrid membrane–based methodology of electrodialysis (ED) with ultrafiltration (UF) to recover the IL [Bmim]Br (1-butyl-3-methylimidazolium bromide) after biomass fractionation, in order to avoid the energy-extensive distilling process [91]. The result showed that the highest overall IL recovery ratio reached 75.2% with the current efficiency of the ED process approaching 79.1%. The specific energy consumption was about 514.1 g/kw·h in this process.

## 4. Pretreatment of Biomass for Enzymatic Saccharification

A suitable pretreatment process for enzymatic saccharification involves: (1) disrupting hydrogen bonds in crystalline cellulose and raising the porosity and surface area of cellulose to promote its hydrolysis; (2) breaking down the chemical bond and physical tangle between hemicelluloses and lignin to facilitate their separation; (3) avoiding the formation of byproducts that are inhibitory to subsequent processes [108]. Owing to the adjustable dissolving ability of ILs, ILs have been increasingly investigated for biomass pretreatment to improve the subsequent enzymatic saccharification, as an alternative technology to the conventional pretreatment methods [109]. After dissolution and regeneration, partial lignin and hemicelluloses were removed from biomass, the degree of crystallinity of the cellulose was reduced and the porosity of the cellulose was increased [110]. All these changes in the compositions and morphology may improve the efficiency of enzymatic saccharification.

### 4.1. Effect of Ionic Liquids

Doherty et al. found that the β parameter of ILs correlates well with the removal of lignin, the reduction of cellulose crystallinity and the improvement of glucose yield [111]. For instance, pretreatment of maple wood flour with [Bmim]Ac (β > 1.0) removed >32% of lignin wood, reduced the cellulose crystallinity from 66% to 41%, and increased the glucose yield from 4.5% to 65%. In contrast, only 19% of the wood flour’s lignin was removed and the decrease in crystallinity and the improvement of the sugar yield were not observed when the biomass was treated with [Bmim][MeSO_4_] (β = 0.60). The IL [Emim]Ac was found to remove more lignin from wood flour and straw than [Bmim]Cl, thus leading to a higher increase of the enzymatic saccharification efficiency [75,112]. A similar phenomenon was also observed for cholinium-based ionic liquids. Sun et al. compared the pretreatment efficiency of four ILs, including cholinium lysinate ([Ch][Lys]), cholinium acetate ([Ch][Ac]), 1-ethyl-3-methylimidazolium lysinate ([Emim][Lys]) and [Emim]Ac [113]. It was observed that pretreatment with ILs containing [Lys]^‑^ anions results in higher lignin removal (70%–80% vs. 16%–50%) and glucose yields (78%–96% vs. 56%–90%) than [Emim]Ac. The efficiency of lignin removal and sugar yield was found to be strongly correlated with the β parameter, while a correlation between the β parameter and the decrement of the crystallinity of the treated biomass was not observed. An et al. investigated the effect of residual lignin on the subsequent enzymatic hydrolysis of biomass treated by cholinium-based ILs [114]. They found the presence of residual lignin has a significantly negative impact on the enzymatic hydrolysis of cellulose. Uju et al. investigated the potential of 1-hexylpyridinium chloride ([Hpy]Cl) to pretreat microcrystalline cellulose and bagasse [115]. They found that [Hpy]Cl pretreatment for an extended duration (180 min) released mono- and disaccharides without using cellulase enzymes, as is different with [Emim]OAc pretreatment.

Improving the compatibility of ILs with enzymes is very important for the optimization of IL-based pretreatment technology [116,117]. The presence of [Bmim]Cl, even at very low concentrations, resulted in the deactivation of cellulases, seriously limiting its practical applications. The IL [Emim]Ac was found to be more compatible with a commercially available cellulase mixture than [Bmim]Cl. Wang et al. found that the cellulases mixture can retain high activity to hydrolyze cellulose in the presence of [Emim]Ac when its concentration is less than 15% [118]. Auxenfans et al. demonstrated that [C_2_mim][MeO(H)PO_2_] is a good compatible IL in which enzymes are not deactivated at concentrations up to a 30% (*v*/*v*) [119].

The high cost of imidazolium cations, which are derived from chemicals synthesized from petroleum sources, is one major obstacle limiting the large-scale industrial application of imidazolium-based ILs for biomass pretreatment. Developing novel ionic liquids based on cations derived from renewable sources seems to be a promising approach to solve this problem. Socha et al. prepared a series of tertiary amine*–*based ILs using aromatic aldehydes, including vanillin, *p*-anisaldehyde and furfural derived from lignin and hemicellulose as starting materials [120]. Two biomass-derived ionic liquids, including [FurEt_2_NH][H_2_PO_4_] and [*p*-AnisEt_2_NH][H_2_PO_4_], showed excellent performance for biomass pretreatment, providing 90% and 96% of total possible glucose and 70% and 76% of total possible xylose, respectively, after enzymatic saccharification of the treated biomass. Although the lignin removal efficiency of these ionic liquids was lower than [Emim]Ac, the final yields of sugars were comparable with the latter. Other biomass-derived ILs, including cholinium glycine ([Ch][Gly]), cholinium alanine ([Ch] [Ala]), cholinium serine ([Ch][Ser]), cholinium threonine ([Ch][Thr]), cholinium methionine ([Ch][Met]), cholinium proline ([Ch][Pro]), and cholinium phenylalanine ([Ch][Phe]), were investigated for biomass pretreatment by Hou et al. [121]. They found these ILs are also very capable of improving the saccharification efficiency of biomass. Gschwend et al. reported that protic ionic liquids synthesized by an accurate combination of aqueous acid and amine base are effective for the deconstruction of lignocellulosic biomass, yielding a purified lignin and leaving a cellulose-rich pulp ready for saccharification [122]. The cost of prtreatment could be consideraly reduced by synthesizing protic ionic liquids containing the hydrogen sulfate [HSO_4_]^−^ anion using inexpensive feedstocks such as sulfuric acid and simple amines [123,124]. Although the saccharification yields were lower than the benchmark system, [Emim]Ac, these ionic liquids were promising to compete with the cheapest pretreatment chemicals, such as ammonia, owing to their low cost [124,125]. Da Costa Lopes et al. investigated the pretreatment of wheat straw with different ILs, including [bmim][HSO_4_], 1-butyl-3-methylimidazolium thiocyanate ([bmim][SCN]), and 1-butyl-3-methylimidazolium dicyanamide ([bmim][N(CN)_2_]) [126,127,128]. They found that only [bmim][HSO_4_] could achieve a macroscopic complete dissolution of wheat straw during pretreatment. A high-purity lignin-rich material was obtained with [bmim][SCN], while a high-purity carbohydrate-rich fraction was produced with [bmim][N(CN)_2_].

### 4.2. Effect of Biomass

The biomass species, size and loading could also greatly influence the pretreatment, as well as the hydrolysis processes, and then impact the whole biorefinery process [129]. Aid et al. investigated the effect of wood species on the pretreatment efficiency of ILs, using spruce, birch, pine and winter wheat straw as feedstock [130]. They found that the highest glucose yield (12.07 mmol/L) was obtained in the case of spruce. Perez-Pimienta investigated the effects of calcium oxalate on biomass pretreatment and saccharification using agave bagasse (AGB) as a model material due to its natural high levels of CaOX [108]. To understand the physicochemical changes in the process of biomass pretreatment, both raw AGB and CaOX-extracted agave bagasse (EAB) were treated by ionic liquid [Emim]Cl and alkaline hydrogen peroxide (AHP). Their results showed that the IL pretreatment achieved a higher sugar yield and lower crystallinity than the latter. Ravanal et al. performed a comparison of different types of pretreatment on the enzymatic saccharification of *Macrocystis pyrifera* [131]. They found that the use of ionic liquids DBU–MEA–SO_2_–SIL and [DBNH][OAc] as pretreatments was relatively efficient for the liberation of uronic acid in the saccharification step from alginate, the main constituent of *Macrocystis pyrifera* (60.6 wt. %).

### 4.3. Effect of Co-Solvent

Except for pure ionic liquid, IL-based solvent systems were also widely investigated for the pretreatment of biomass. Weerachanchai et al. studied the effect of organic solvent on the pretreatment of biomass [132]. They found that the pretreatment efficacy of the solvent system consisting of 40–60 vol % of dimethylacetamide (DMA) and [Emim]Ac is comparable with pure [Emim]Ac. On the contrary, the pretreatment efficacy of [Emim]Ac/ethanolamine (60/40 vol %) and [Bmim]Cl/ethanolamine (60/40 vol %) was remarkably higher than that of the corresponding ILs. After pretreatment with [Bmim]Cl/ethanolamine (60/40 vol %), the sugar conversion can reach 90 wt. %, which is 25%–39% higher than that obtained with [Bmim]Cl. At the same time, the partial displacement of ILs with ethanolamine has others advantages, including low viscosity, high loading of cellulose, and low cost. Parthasarathi et al. reported that the inexpensive IL comprised of tetrabutylammonium and hydroxide [OH]^−^ ions can be used to pretreat biomass at very mild processing conditions (50 °C) [133]. High glucose yields (~95%) were achieved by enzymatic saccharification of the biomass after pretreatment. Compared with conventional IL-based pretreatment technology, the energy inputs in the pretreatment unit operation were reduced by more than 75% owing to the mild operating conditions. Chang et al. reported that adding some surfactants to ionic liquid [Bmim]Cl can significantly improve the pretreatment efficiency compared with the [Bmim]Cl-only pretreatment [134]. The removal of lignin was increased by 49.48% through adding 1% sodium dodecyl sulfate (SDS). Moreover, the cellulose crystallinity was reduced and the surface morphology of the rice straw, including the porosity and surface area, was significantly changed after the surfactant-assisted IL pretreatment.

### 4.4. Comparison of Different Pretreatment Methods

Perez-Pimienta et al. performed a comparative assessment of three pretreatment methods, including ammonia fiber expansion (AFEX™), autohydrolysis (AH) and IL pretreatment [135]. They found that almost all the carbohydrates in the biomass were preserved after AFEX pretreatment, whereas 62.4% of xylan was removed by AH and 25% of lignin was fractionated into the wash streams by IL. Nguyen et al. investigated the combined use of ammonia and IL [Emim]Ac for the pretreatment of rice straw [136]. They found this combined pretreatment could obtain 82% of the cellulose recovery with 97% of the glucose yield, which are significantly higher than values of the individual ammonia (84% and 52%) or ILs treatments (79% and 76%). In addition, the usage of an enzyme and the incubation time can be reduced. Excellent convesion rates of glucan-to-glucose (between 75% and 97%) were obtained after the enzymatic hydrolysis of substrates treated with ionic liquids, including DBU–MEA–SO_2_ and DBU–MEA–CO_2_, [Amim][HCOO] and [AMMorp]Ac, while the convesion rates were between 13%–77% for the combined acid treatment and enzymatic hydrolysis [137]. In addition, to compare the efficiency of enzymatic saccharification, a systematic techno-economic analysis of different pretemant methods is necessary to obtain the optimized biomass processing technology.

### 4.5. Process Considerations

Producing concentrated sugars and minimizing water usage are key elements in the economics and environmental sustainability of advanced biofuels. Conventional pretreatment processes that require a water-washing step result in losses of fermentable sugars and generate large volumes of wastewater or solid waste. The strategy of simultaneous saccharification and fermentation (SSF) as well as separate hydrolysis and fermentation (SHF) was compared by Lienqueo et al. [138]. They found that the SHF process yielded 0.134 g ethanol/g glucose (26.3 wt. % of the theoretical yield), while the SSF process yielded 0.173 g ethanol/g glucose (33.9 wt. % of the theoretical yield) within 24 h of fermentation.

**Figure 3:**
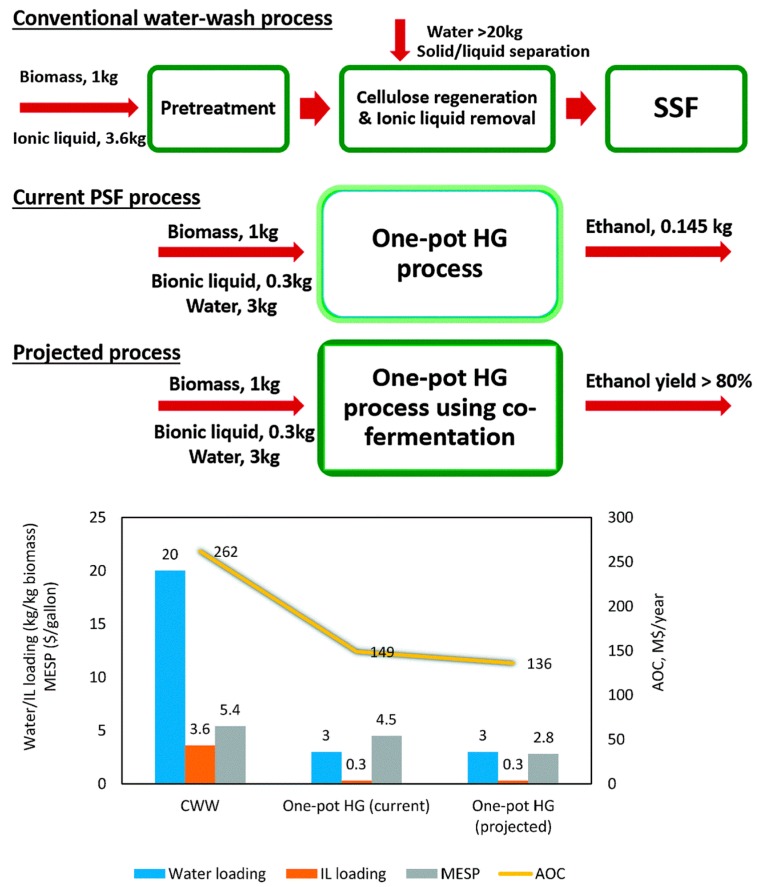
Comparison of three scenarios in terms of water loading, ionic liquid (IL) loading, annual operating costs (AOC), and minimum ethanol selling price (MESP). Scenario 1. The conventional ionic liquid process, including a water-washing step before simultaneous saccharification and fermentation (SSF); Scenario 2. One-pot high-gravity (HG) PSF (pretreatment, saccharification, and fermentation) configuration for ethanol production from glucose; Scenario 3. A projected system based on the current one-pot high-gravity configuration plus co-fermentation of ethanol from both glucose and xylose. Adapted from Reference [139] with permission from The Royal Society of Chemistry.

Xu et al. developed a one-pot biomass processing scheme which integrates IL pretreatment, enzymatic saccharification and yeast fermentation together for the direct production of concentrated fermentable sugars and high-titer cellulosic ethanol, as shown in Figure 3 [139]. In this scheme, the use of dilute bio-derived ionic liquids (bionic liquids) makes it possible to directly hydrolyze the biomass after pretreatment without a wash-up and separation step. After adjusting the pH, the obtained biomass solution was directly subjected to enzymatic saccharification and microbial fermentation using commercially available enzyme mixtures and fermentation hosts. High-gravity bioethanol (equivalent to an overall yield of 74.8% on a glucose basis) can be produced from the one-pot process. This process could reduce the amount of ionic liquid required by ~90% through the use of dilute bionic liquids. Meanwhile, the water usage and waste generation was reduced by ~85%, compared with conventional pretreatment processes that require a water-washing step. In turn, these improvements can reduce the net electricity use, greenhouse gas–intensive chemical inputs for wastewater treatment, and waste generation. A preliminary technoeconomic analysis indicated that an overall 40% reduction in the cost is achieved using this technology.

The one-pot high-gravity process was further integrated by Sun et al. through using CO_2_ as a reversible method to adjust the pH which enabled the direct enzymatic saccharification of treated biomass without separation and purification [140]. Through the integrated process, high yields of fermentable sugars and ethanol can be obtained using commercially available enzyme mixtures and fermentation hosts. A preliminary technoeconomic analysis suggested this approach can reduce the production costs by 50%–65%, compared with the conventional IL-based pretreatment.

Except for ethanol, the production of other advanced biofuels can be also improved by pretreatment of the biomass with ILs. For instance, Liszka et al. reported a highly efficient one-pot process to produce isopentenol from biomass based on switchable ionic liquids [141]. The switchable ionic liquids can transform from the basic form which is capable of pretreating biomass to its slightly acid form that is compatible with enzymatic saccharification and subsequent microbial fermentation. The unique property of the switchable ILs enables the direct enzymatic saccharification and fermentation of treated biomass in a one-pot process without removing the ionic liquid or inhibitors prior to fermentation. After pretreatment, a 90% conversion of lignocellulosic biomass to fermentable sugars was achieved using commercially available enzyme mixtures. Isopentenol can be obtained through fermentation of the crude hydrolysate using *E. coli*. This integrated process eliminated the need for intermediate washing and/or separation steps. Compared with the biorefinery process based on traditional ILs ([Emim]Ac), the minimum selling price of the final products can be reduced by more than $1 gal^−1^. In addition, Gao et al. reported that ionic liquid pretreatment could also improve the efficiency of the anaerobic digestion of lignocellulosic biomass [142].

## 5. Conclusions and Perspectives

This article gives an overview on the applications of ionic liquids (ILs) and IL-based solvent systems in the pretreatment of lignocellulosic biomass, including the dissolution of biomass in ILs and IL-based solvent systems, the fractionation of biomass using ILs and IL-based solvent systems as solvents and the enzymatic saccharification of pretreated biomass. A reasonable design of ILs and the use of IL-based solvent systems have been demonstrated to be effective to improve the dissolution, pretreatment and fractionation efficiency; to reduce the cost of ILs; and to improve their compatibility with enzymes. As a consequence, the operation process can be simplified, and then the input and cost can be reduced. To develop an integrated biorefinery process which could convert biomass into fuels, chemicals and materials in an affordable, scalable and sustainable way, more attention should be paid to the careful selection of ILs, catalysts and processing conditions as well as the optimization of biorefinery processes. The role of ILs and related solvents in the pretreatment of lignocellulosic biomass should be deeply understood to design novel, tailor-made ILs and solvent systems to further improve the pretreatment efficiency. The compatibility between upstream and downstream processing should also be improved to achieve the full utilization of the biomass. At the same time, the relative technologies, including synthesis, separation and purification, microbiological cultivation, production of enzymes and waste treatment, should also be studied in depth to underpin the development of biorefinery technologies. In addition, systematic technoeconomic analysis and environmental impact analysis should be performed to rationalize these processes.

## Figures and Tables

**Table 1:** Solubility of cellulose in ILs and IL-based solvent systems.

Solvents	Cellulose	Dissolution Conditions	Solubility (%)	Reference
[Emim]Cl	Mcc	100 °C	10	[9]
[C_3_mim]Cl	Mcc	100 °C	0.5	[9]
[Bmim]Cl	Mcc	100 °C	20	[9]
[Emim]Ac	Mcc	100 °C	8	[9]
[MeOC_3_P][MeOAc]	Avicel	100 °C	36	[33]
[AC_2_P][MeOAc]	Avicel	100 °C	31	[33]
[Bmim]Ac/DMSO	Mcc	25 °C	15.0	[39]
[Bmim]Ac	Mcc	50 °C	11.5	[39]
[Bmim]Ac/LiAc	Mcc	50 °C	16.0	[42]
[Amim]Cl	Pulp(650)	80 °C	14.5	[46]
[Amim]Cl	Mcc-NaOH	100 °C	3.5	[46]
[AC_2_im]Cl	Mcc-NaOH	100 °C	5.4	[46]
[AMMorp]Ac	Mcc	120 °C	25–30	[47]

**Table 2:** Solubility of lignin in ILs and IL-based solvent systems.

Solvent	Dissolution Conditions	Time	Lignin (%)	Cellulose (%)	Xylan (%)	Reference
[Py][For]	75 °C	1 h	70	<1		[48]
[Py][Pro]	75 °C	1 h	55			[48]
[Py][Ac]	75 °C	1 h	64			[48]
[Mmim][MeSO_4_]	80 °C	24 h	50			[49]
[Bmim][CF_3_SO_3_]	80 °C	24 h	50			[49]
[Py][Ac]	90 °C	24 h	>50	0.12 ± 0.03	0.82 ± 0.00	[34]
[Mim][Ac]	90 °C	24 h	>50	0.20 ± 0.05	5.60 ± 0.77	[34]
[Pyrr][Ac]	90 °C	24 h	>50	0.79 ± 0.04	>15	[34]
GVL/[Bmim]Ac	30 °C		20.9			[53]
GVL/[Bmim]Ac	60 °C		28.0			[53]
GVL/[Amim]Cl	30 °C		13.4			[53]
GVL/[Amim]Cl	60 °C		26.6			[53]
[Emim]Ac/water (wt:wt 70:30)	60 °C		45			[54]

**Table 3:** Separation and recovery of lignin from biomass using ILs and IL-based solvent systems.

Biomass	Ionic Liquid	Condition	Anti-Solvent	Yield of Lignin	Reference
Corn stock	[Pyrr][Ac]	90 °C, 24 h		70%	[34]
Pinus radiata	[C_2_mim]Ace	100 °C, 2 h	Acetone	43%	[71]
Pinus radiata	[C_4_mim]Ace	100 °C, 2 h	Acetone	38%	[71]
Pinus radiata	[C_4_mim]Ace/DMSO	100 °C, 2 h	Acetone	58%	[71]
Pine	[Emim]Ac	110 °C, 16 h	Acetone/water	31%	[74]
Maple	[Emim]Ac	130 °C, 1.5 h	0.1 M NaOH	63%	[75]
Maple	[Emim]Ac	80 °C, 24 h	0.1 M NaOH	51%	[75]
Maple	[Mmim][MeSO_4_]	80 °C, 24 h	0.1 M NaOH	9%	[75]
Maple	[Bmim][CF_3_SO_3_]	80 °C, 24 h	0.1 M NaOH	6%	[75]
Bagasse	[C_2_mim][ABS]	180 °C, 2 h	0.1 M NaOH	78%	[76]
Bagasse	[C_2_mim][ABS]	190 °C, 2 h	0.1 M NaOH	118%	[76]
Bagasse	[C_2_mim][ABS]	190 °C, 1.5 h	0.1 M NaOH	97%	[76]
